# Protein fishing from single live cells

**DOI:** 10.1186/s12951-018-0395-5

**Published:** 2018-09-11

**Authors:** Elaheh Shekaramiz, Rupak Doshi, H. Kumar Wickramasinghe

**Affiliations:** 1 Department of Biomedical Engineering, University of California Irvine, Irvine, CA USA; 2 Department of Electrical Engineering, University of California Irvine, Irvine, CA USA; 3 InhibRx LLP, 11025 N Torrey Pines Rd, #200, La Jolla, CA 92037 USA

**Keywords:** Proteomics, Nanopipette, Single cell analysis, Live cell protein detection

## Abstract

Intracellular protein and proteomic studies using mass spectrometry, imaging microscopy, flow cytometry, or western blotting techniques require genetic manipulation, cell permeabilization, and/or cell lysis. We present a biophysical method that employs a nanoaspirator to ‘fish’ native cytoplasmic or nuclear proteins from single mammalian cells, without compromising cell viability, followed by *ex cellulo* quantitative detection. Our work paves the way for spatiotemporally-controlled, quantitative, live, single-cell proteomics.

**Electronic supplementary material:**

The online version of this article (10.1186/s12951-018-0395-5) contains supplementary material, which is available to authorized users.

## Introduction

Intracellular proteins have historically been detected and quantified using western blot, in which a population of cells is lysed, the contents are separated by gel electrophoresis, followed by detection using antibodies that target the specific protein(s)-of-interest [1]. While this has been an extremely successful technique used for decades, its detection scale is limited to a small set of proteins and cellular lysis prevents longitudinal studies at the single cell level [2]. Mass spectrometry-based protein detection also requires cell lysis, although it overcomes the scaling limit of western blot by offering unprecedented resolution of proteins and high-content proteomics analysis [3].

Recent interest has turned to protein detection techniques that are more amenable to studying live cells [4–6]. Intracellular flow cytometry staining can circumvent total cell lysis [7]. However, the technique requires cell fixation to stabilize intracellular proteins, followed by cell permeabilization to allow for the entry of detection antibodies, hindering longitudinal studies [8, 9]. Moreover, most primary antibody reagents available from commercial sources have not been tested and validated [10] for intracellular flow cytometry, which makes assay development a tedious task. Imaging microscopy of live cells has achieved super-resolution with tremendous spatio-temporal control [11], but requires the cloning of fluorescent proteins or epitope tags onto the protein(s)-of-interest, through over-expression plasmids or genetic knock-ins, negating native proteomic studies.

We are interested in methods that allow the scalable detection of native proteins and proteomes from single and live mammalian cells in real-time, without requiring: [1] cell lysis, [2] fixation/permeabilization, or [3] cloning. A small number of techniques have emerged in recent times that fit these criteria [12–15]. Reports from Singhal et al. [12] and Actis et al. [13] are excellent technological progresses, but their methodologies were not developed for protein studies. Guillaume-Gentil et al. [14] used fluidic force microscopy to extract 3000 fL of the cytoplasm of a live HeLa cell, and successfully detected activity of native β-gal present in the extract. Although, the authors showed that cellular survival was unaffected despite extracting up to 90% of the cytoplasm, manipulations of such large volumes of a cell could drastically alter native proteomic signatures and undermine single cell analysis. Cao et al. [15] developed a non-destructive intracellular protein extraction platform, where cells are cultured on a nanostraw-embedded membrane, and briefly electroporated to release cellular contents into a sampling buffer for analysis. The technique allows for longitudinal sampling of proteins and mRNA from the cytoplasm of single, or a small population of cells, without compromising cell viability. However, with the nanostraws being immobile themselves, this technique offers limited spatial control over sampling from sub-cellular organelles. Furthermore, because the sampled cytoplasm and biomolecules are diluted in extraction buffer, an additional processing step, such as isotachophoresis-mediated sample pre-concentration, is required prior to proceeding with protein analysis. Other methods include the employment of host–guest systems for selective isolation of specific proteins from a cell using bait chemistry [16], but these methods lack proteomic scalability.

Here, we present a nanoaspirator-based platform with significant improvements over the above-described methods. Spatiotemporally-controlled native protein extraction and direct, quantitative detection from 20 to 50 fL of a single, live mammalian cell are shown, in a biochemical environment-responsive experimental setting.

## Materials and methods

### Fabrication of integrated electrowetting nanoaspirators

Nanoaspirators were fabricated from borosilicate glass capillaries (Sutter Instrument, Novato, CA) using a P-97 puller (Sutter Instrument, Novato, CA). Integrated Electrowetting nanoaspirators were sputter coated with 10 nm layer of iridium followed by 20 nm platinum on one side. The probes were oxygen plasma treated at a power of 100 W for 10 min before the experiment. Nanoaspirators were filled with a solution of 1,2-dichloroethane (DCE) containing 10 mM tetrahexyl ammonium bromide. A silver wire coated with AgCl was then inserted into the barrel of the nanoaspirators.

### Cell culture

Cells were cultured in 90% Dulbecco’s modified Eagle’s medium (DMEM) with 10% FBS (fetal bovine serum), at 37 °C with 5% CO_2_. NIH3t3 and HeLa cells were purchased from ATCC and used at 70% confluency for each experiment. Cell viability was monitored using the trypan blue test.

### Actinomycin D treatment

Actinomycin D (2 mg/mL) stock from Sigma-Aldrich was diluted in 90% DMEM, with 10% FBS to a final concentration of 50 nM. HeLa Cells were treated with 50 nM of actinomycin D 24 h post-passage and were probed 24 h after treatment.

### Cytoplasmic and nuclear extraction

To locate where the proteins were released, grids were created on top of coverslips. TEM grids were purchased from SPI and were placed on top of coverslip. 20 nm of Iridium was sputter coated on top of it. The coverslips were coated with 2% APTES [(3-aminopropyl) triethoxysilane)] in ethanol overnight. They were washed with ethanol and DI water after the coating and were used for protein extraction. The experiments with the anti-actin antibody tagged FITC were performed by simply picking up and releasing the different volumes of antibody on top of coverslip and measuring the raw integrated density signal using ImageJ software. However, the standard curve for the actin antigen was performed by picking up various volumes of actin molecules and performing the staining procedure on top of APTES coated coverslips.

### Deposition of extracts

In order to facilitate localization of the deposited biomolecules, grids were created on top of coverslips. TEM grids (SPI supplies) were placed on top of coverslip. 20 nm of Iridium was sputter-coated on top. These coverslips were further coated with 2% APTES in ethanol overnight, followed by ethanol and DI water washes prior to use. Deposition was performed by enabling physical contact between the nanoaspirator tip and the coverslip, resulting in disruption of the tip’s glass surface and release of the extracted contents onto a tight, sub-µm-sized spot on the grid, visible under white light.

### Antibody staining

Specific detection and quantification of proteins in the deposited cellular extracts were performed using fluorescent antibody conjugates. FITC-anti-β-actin monoclonal antibody (Clone AC-15, AbCam) and APC-anti-p53 monoclonal antibody (Clone 184727, R&D systems) were used in our experiments. After deposition of the extracted cytoplasm/nucleoplasm on top of APTES coated coverslips, 1% BSA in PBS was used for 1 h at room temperature for blocking. The coverslips were then washed three times with 1× PBS. Antibodies were added and incubated for 1 h at 4 °C, washed with 1× PBS three times and detected through fluorescence imaging using Photometric Evolve 512 Delta EMCCD camera. The standard curve for the β-actin antigen was performed by sampling-and-depositing various calculated volumes containing set numbers of β-actin molecules on APTES-coated coverslips, performing the staining with the FITC-anti-β-actin antibody as described, on the coverslips, and measuring the raw integrated density signal using the ImageJ software.

## Results and discussion

**Fig. 1:**
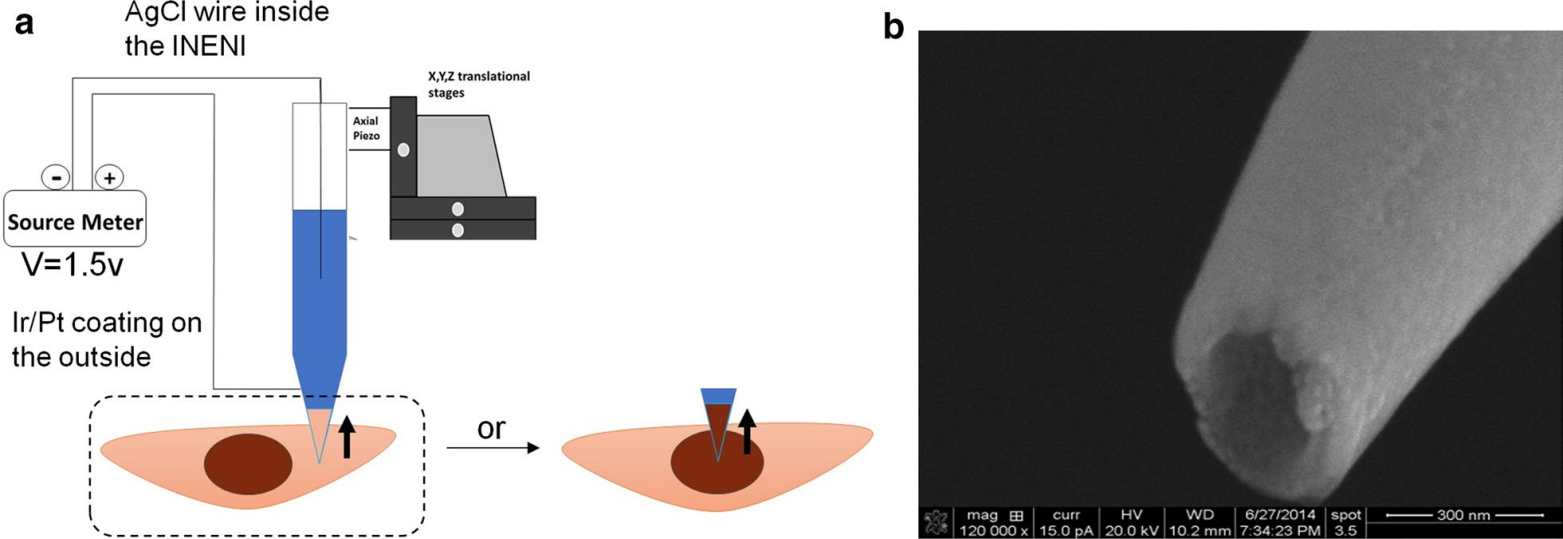
Schematics of the nanoaspirator setup. **a** The aspiration set up is comprised of X, Y, Z translation stages for course movement and a peizo actuator for fine movements. The nanoaspirator is mounted to the piezo actuator. The inner electrode and the outer Ir/Pt coated electrode are connected to a sourcemeter for voltage applications. **b** SEM image of the nanoaspirator tip

We have previously used an integrated electrowetting nanoinjector (INENI) fabricated in our lab for single cell transfections, where we injected plasmid DNA into mammalian cells, while maintaining near-complete cell viability [17]. Our nanoaspirator design, shown in Fig. 1, has been built off of our INENI work [17]. The nanopipette was calibrated for handling femtoliter (fL) volumes of solution, as described before [17]. To quantify the uptake of fluid volume with respect to applied potential difference, different voltages were applied and the corresponding increase in fluid height within the nanopipette was measured using the ImageJ software, and a transmission electron microscopy (TEM) reference grid [17]. The radius corresponding to the height of liquid was calculated based on the angle of the cone, which from SEM images, was measured to be 4.3°, giving an average radius of 70.2 nm [17]. Details of the mathematical theory and data supporting our model have been described in our previous work [17].

Initial method setup involved the aspiration and release of pure, fluorescein-conjugated BSA (bovine serum albumin) solutions in vitro (Additional file 1: Figure S1A). The fluorescence could be tracked in the nanoaspirator and visualized upon deposition onto a coverslip (Additional file 1: Figure S1A). To establish nanoaspiration from cells, the z-fine movement piezo was calibrated to precisely enter single cells, which was monitored through real-time measurements of perturbations in current upon cellular (cytoplasmic or nuclear) entry (Additional file 1: Figure S1B). NIH 3t3 cells were electroporated with a plasmid expressing GFP (Additional file 1: Figure S1C), and after 24 h, GFP-positive-cells were successfully used for nanoextraction of cytoplasm containing GFP, followed by its deposition, and fluorescence imaging (Additional file 1: Figure S1C). Importantly, we saw the maintenance of complete cell viability after cytoplasmic extractions in our trypan blue tests, performed on one and the same cell post-nanoextraction (Additional file 1: Figure S1A–C).

**Fig. 2:**
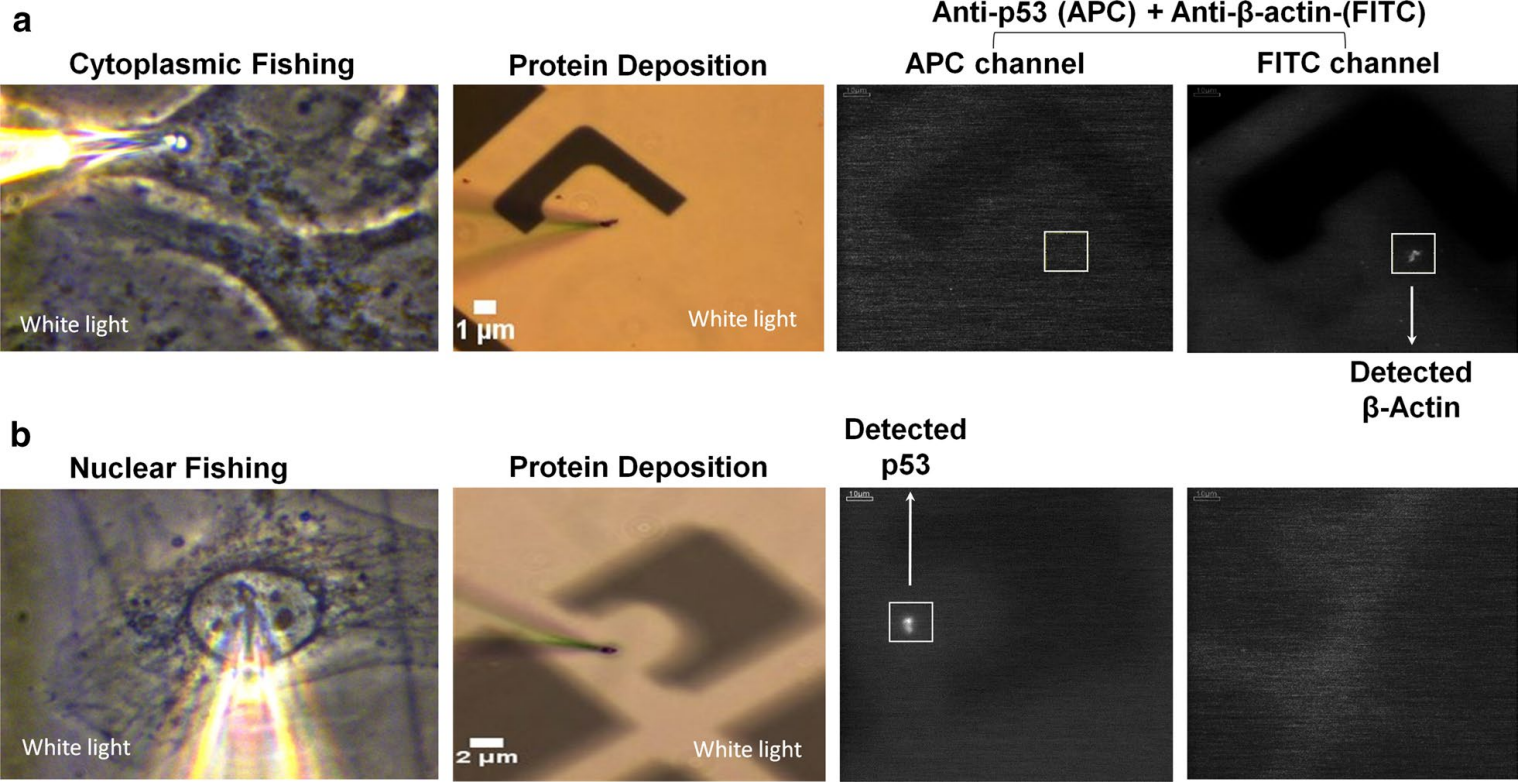
Protein fishing from HeLa cells. Sampling of HeLa cells 24 h after actinomycin D treatment. **a** Cytoplasmic or **b** nuclear extract was deposited on grid-marked, APTES-coated coverslips, followed by protein detection using a cocktail of anti-β-actin-FITC and anti-p53-APC. The coverslips were imaged under the FITC and APC filter channels, sequentially

In order to show that we can detect environment-specific native protein signatures of a cell, we chose a model system where the upregulation of a stress-response protein in a cell has been previously studied using western blot. The cervical carcinoma cell line, HeLa, has been shown to have suppressed expression of the tumor-suppressor gene, p53 [18]. Heitanen et al. [19] showed that p53 gets upregulated upon the addition of anticancer drug compounds, such as actinomycin D to cells in culture. We cultured HeLa cells and treated them with actinomycin D similarly, and proceeded with proteomic extraction and detection of p53, in reference to the housekeeping gene, β-actin (Fig. 2).

In our experiments using cytoplasmic extractions, we were unable to detect any p53 in the untreated or treated samples 24 h after treatment, while β-actin was detected successfully (Fig. 2a). Consistent with Hietanen et al. [19] p53 was successfully detected in the nucleus of actinomycin-treated HeLa cells, 24 h post-treatment, wherein, no β-actin was detectable (Fig. 2b). Interestingly, we were able to detect both, p53 and β-actin, in the cytoplasm 36 h after treatment (Additional file 1: Table S1), which sheds light on the nucleocytoplasmic shuttling dynamics of p53 [20] under the cell culture conditions used in our assays. It is noteworthy that nanoaspirations from the nucleus of cells resulted in an estimated 85.7% cell viability (Additional file 1: Figure S2B, C). It is also important to note that the absence of nonspecific cross-staining, i.e. no p53 in the cytoplasm and no β-actin in the nucleoplasm at the 24 h mark (Fig. 2a, b), provides strong evidence for the specificity of the detection antibodies used in our assays.

**Fig. 3:**
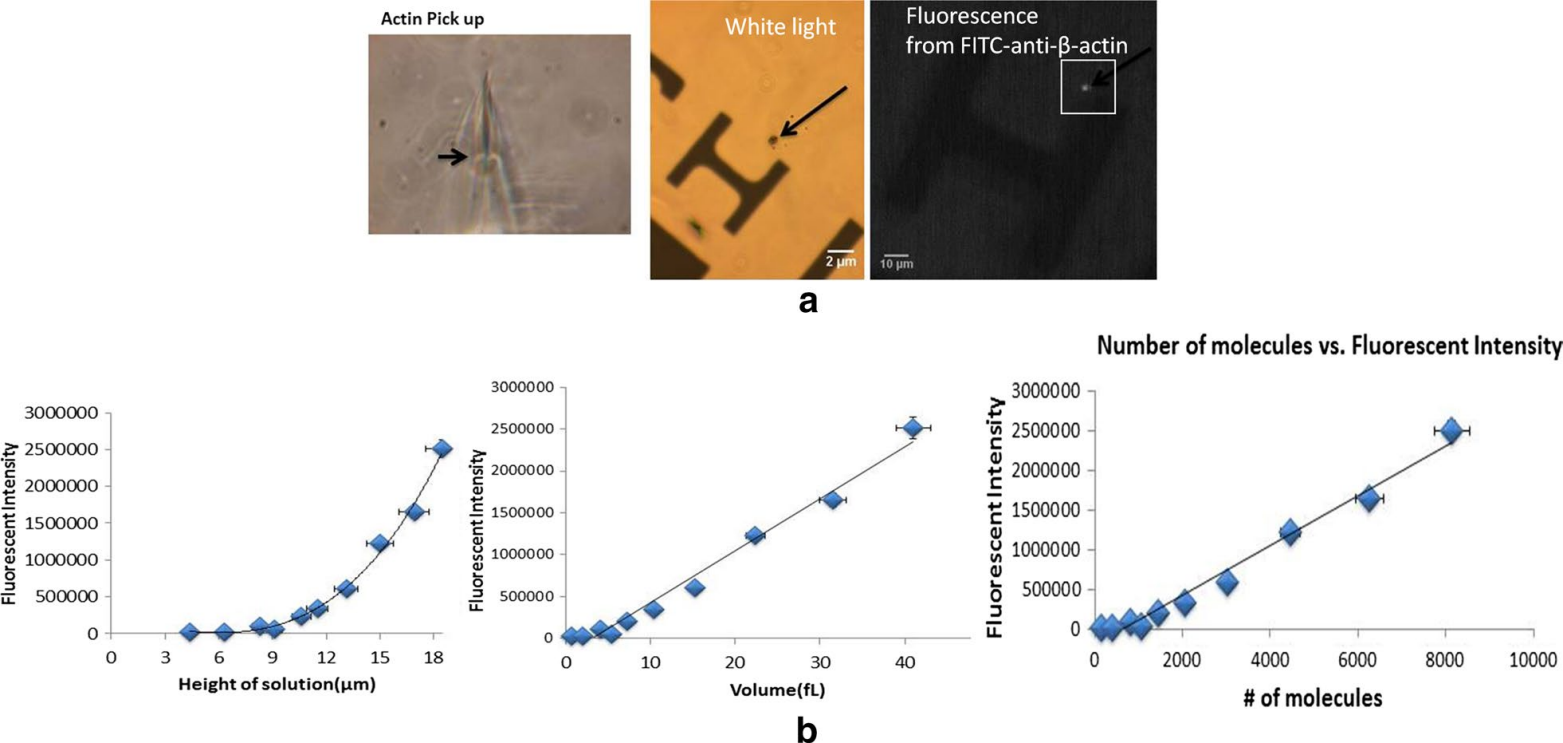
Standard curve generation for β-actin. **a** A standard curve for β-actin was generated by the aspiration-and-deposition of different volumes of pure β-actin solution on a coverslip, and **b** correlating the number of spotted molecules (gauged from the height of solution in the nanopipette) with the fluorescence intensity of FITC-anti-β-actin, which was used to stain the spot

**Fig. 4:**
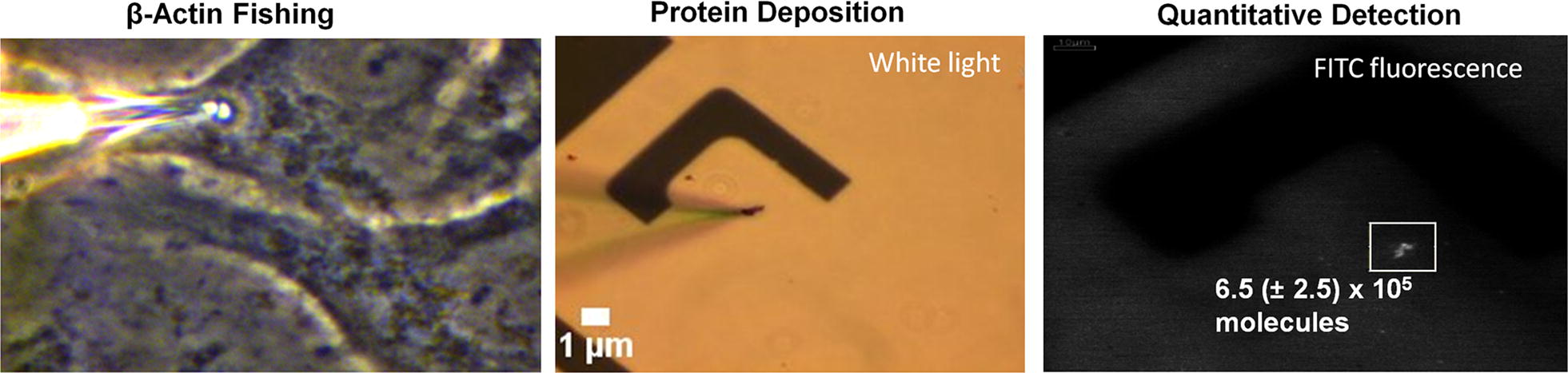
β-Actin quantification from NIH 3T3 extracts. Aspiration and quantification of β-actin from the cytoplasm of a single NIH 3T3 cell. Fluorescence from the spot was used to extrapolate the number of molecules from the standard curve shown in Fig. 3. The total number of molecules in the 20–50 fL extract was calculated to be 6.5 ± 2.5 × 10^5^ (mean ± range, N = 4), which scales to 6.5 ± 2.5 × 10^7^ for a total cell volume of ~ 2000 fL

Next, we proceeded to demonstrate that the cellular extracts deposited on coverslips, using our nanoaspirator setup, can be used to quantify native protein levels. For proof-of-concept, we decided to quantify one of the most abundant proteins in the cell, β-actin. In order to obtain a standard curve, pure β-actin protein solutions were aspirated and deposited on APTES-coated coverslips, followed by antibody staining (Fig. 3a). The volumes of each release of β-actin were calculated from the height of liquid in the aspirator, which were extrapolated to the number of molecules, and plotted versus fluorescent intensity of the signal (Fig. 3b). Using this standard curve, we quantified native β-actin amounts in our cytoplasmic extractions of 20–50 fL from NIH 3T3 cells, which was calculated to be 6.5 ± 2.5 × 10^5^ molecules (mean ± range, N = 4) (Fig. 4). Since we sampled only 1% volume of the cell (20–50 fL of the 2000 fL total volume of the cell), we extrapolate the quantified total number of β-actin molecules per cell to 6.5 ± 2.5 × 10^7^. We made three assumptions in this quantification scheme; [1] uniform distribution of this cytoskeletal protein throughout the cytoplasm, [2] FITC-conjugated antibody saturates all β-actin molecules present in the extract and is directly proportional to fluorescence, and [3] that the anti-β-actin antibody binds to pure and cellular β-actin with the same affinity and avidity. Schwanhäusser et al. [21] used metabolic pulse labeling to measure a total of 10^8^ molecules of β-actin inside mouse fibroblast cells, which stands in excellent agreement with our calculated value, thereby, providing strong validation for our single cell, native protein quantification methodology, including its assumptions.

β-Actin is among the most abundantly-expressed proteins in the cell [21]. Naturally, its absolute quantity was at the higher end of our standard curve (Fig. 3). According to the study from Schwanhäusser et al. the median protein copy number in a cell is ~ 5 × 10^4^ [21]. Our standard curve was linear down to ~ 10^3^ molecules (Fig. 3), and with additional improvements through the use of brighter fluorophore-conjugated antibodies and higher-power fluorescence imagers, our detection range should be sufficient to quantify native amounts of a majority of proteins-of-interest from a single cell.

## Conclusion

Based on our results from two different cell lines, mouse NIH3T3 and human HeLa, and 3 proteins, GFP, β-actin and p53, we believe that our platform is generalizable to all mammalian cells and proteins. Since the nanoextraction platform samples cytoplasm or nucleoplasm without biases, the method is only limited by the number and quality of detection antibodies available for the proteins of interest.

While we have shown the quantitative detection of an abundant native protein from 1% of a mammalian cell, we have also shown that the dynamic range of our method is sufficient to detect most of the proteins at their native levels in single cells. Although we have detected two proteins, namely, β-actin and p53, in cellular extracts, several protein panels may be tested on the same coverslip, in principle, limited only by the availability, specificity, and spectral independence of fluorescent detection antibodies. With a global effort towards the development of multiplexed antibody panel arrays to detect several proteins from small sample volumes [22–27], we believe that total proteomic analysis with spatiotemporal control at the single, live cell level is within reach of our technology presented herein.

Previously, we have used a similar nanopipette setup to inject nucleic acid material into single, live cells [17]. Additionally, we have also worked on the detection of transcripts from cellular extracted samples, similar to the protein analysis shown above (unpublished). Together, these tools will create a powerful technology suite to study and manipulate native, single-cell genomes and proteomes of mammalian cell lines, primary cultures, and importantly, precious clinical samples.

## Additional file

**Additional file 1: Figure S1.** Nanoaspiration method development. (A) Fluorescein-tagged-BSA being aspirated and released on top of APTES-coated coverslips. Fluorescence of BSA solutions can be tracked inside the nanoaspirator and on the coverslip, when imaged under the FITC filter channel. (B) Real-time current measurements were used to track the nanopipette’s entry into the cytoplasm and nucleus of cells. Nuclear entry resulted in a greater magnitude of ΔA (i.e. change in current). (C) GFP-encoding plasmid was electroporated into NIH 3T3 cells. 24 h after electroporation, a single, GFP-expressing cell was nanoaspirated and deposited onto a coverslip, followed by FITC channel imaging. **Figure S2.** Cell viability followed by nanoaspiration. (A) Cytoplasmic or (B) nuclear nanoaspiration from HeLa cells was followed by staining of the same cell with a live/dead cell stain, trypan blue. Trypan blue stained or unstained cells look identical, suggesting the maintenance of complete cell viability. (C) Viability percentages were calculated to be 100% and 85.7% for cytoplasmic and nuclear aspirations, respectively. (D) Dead cells that take up the trypan blue stain more readily are shown alongside for comparison purposes. **Table S1.** Protein detection from drug-treated cells. Longitudinal sampling resulting in the positive or negative detection of β-actin and p53 proteins, in cytoplasmic or nuclear extracts from actinomycin D-treated or untreated HeLa cells.

## Abbreviations

DCE: 1,2-dichloroethane
DMEM: Dulbecco’s modified Eagle’s medium
FBS: fetal bovine serum
APTES: (3-aminopropyl) triethoxysilane
INENI: electrowetting nanoinjector
BSA: bovine serum albumin
