# Supplementary material for: Protein fishing from single live cells
Source: J Nanobiotechnology. 2018 Sep 11;16:67. doi: 10.1186/s12951-018-0395-5 (PMC6134770; doi:10.1186/s12951-018-0395-5)
Supplement: Supplementary file 1 — Additional file 1: Figure S1. Nanoaspiration method development. (A) Fluorescein-tagged-BSA being aspirated and released on top of APTES-coated coverslips. Fluorescence of BSA solutions can be tracked inside the nanoaspirator and on the coverslip, when imaged under the FITC filter channel. (B) Real-time current measurements were used to track the nanopipette’s entry into the cytoplasm and nucleus of cells. Nuclear entry resulted in a greater magnitude of ΔA (i.e. change in current). (C) GFP-encoding plasmid was electroporated into NIH 3T3 cells. 24 h after electroporation, a single, GFP-expressing cell was nanoaspirated and deposited onto a coverslip, followed by FITC channel imaging. Figure S2. Cell viability followed by nanoaspiration. (A) Cytoplasmic or (B) nuclear nanoaspiration from HeLa cells was followed by staining of the same cell with a live/dead cell stain, trypan blue. Trypan blue stained or unstained cells look identical, suggesting the maintenance of complete cell viability. (C) Viability percentages were calculated to be 100% and 85.7% for cytoplasmic and nuclear aspirations, respectively. (D) Dead cells that take up the trypan blue stain more readily are shown alongside for comparison purposes. Table S1. Protein detection from drug-treated cells. Longitudinal sampling resulting in the positive or negative detection of β-actin and p53 proteins, in cytoplasmic or nuclear extracts from actinomycin D-treated or untreated HeLa cells. [file 12951_2018_395_MOESM1_ESM.docx]

**Protein Fishing from Single Live Cells**

**Additional file**

**
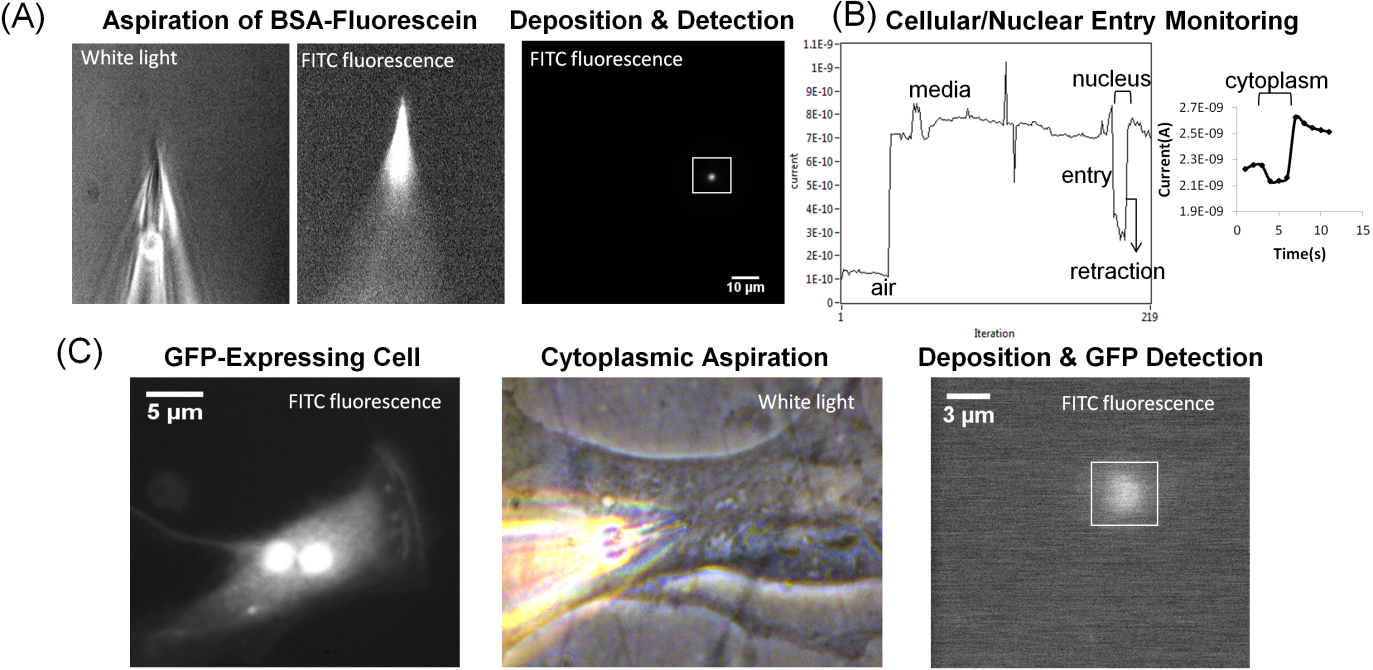
**

**Figure S1: Nanoaspiration method development.** (A) Fluorescein-tagged-BSA being aspirated and released on top of APTES-coated coverslips. Fluorescence of BSA solutions can be tracked inside the nanoaspirator and on the coverslip, when imaged under the FITC filter channel. (B) Real-time current measurements were used to track the nanopipette’s entry into the cytoplasm and nucleus of cells. Nuclear entry resulted in a greater magnitude of ΔA (i.e. change in current). (C) GFP-encoding plasmid was electroporated into NIH 3T3 cells. 24h after electroporation, a single, GFP-expressing cell was nanoaspirated and deposited onto a coverslip, followed by FITC channel imaging.

**
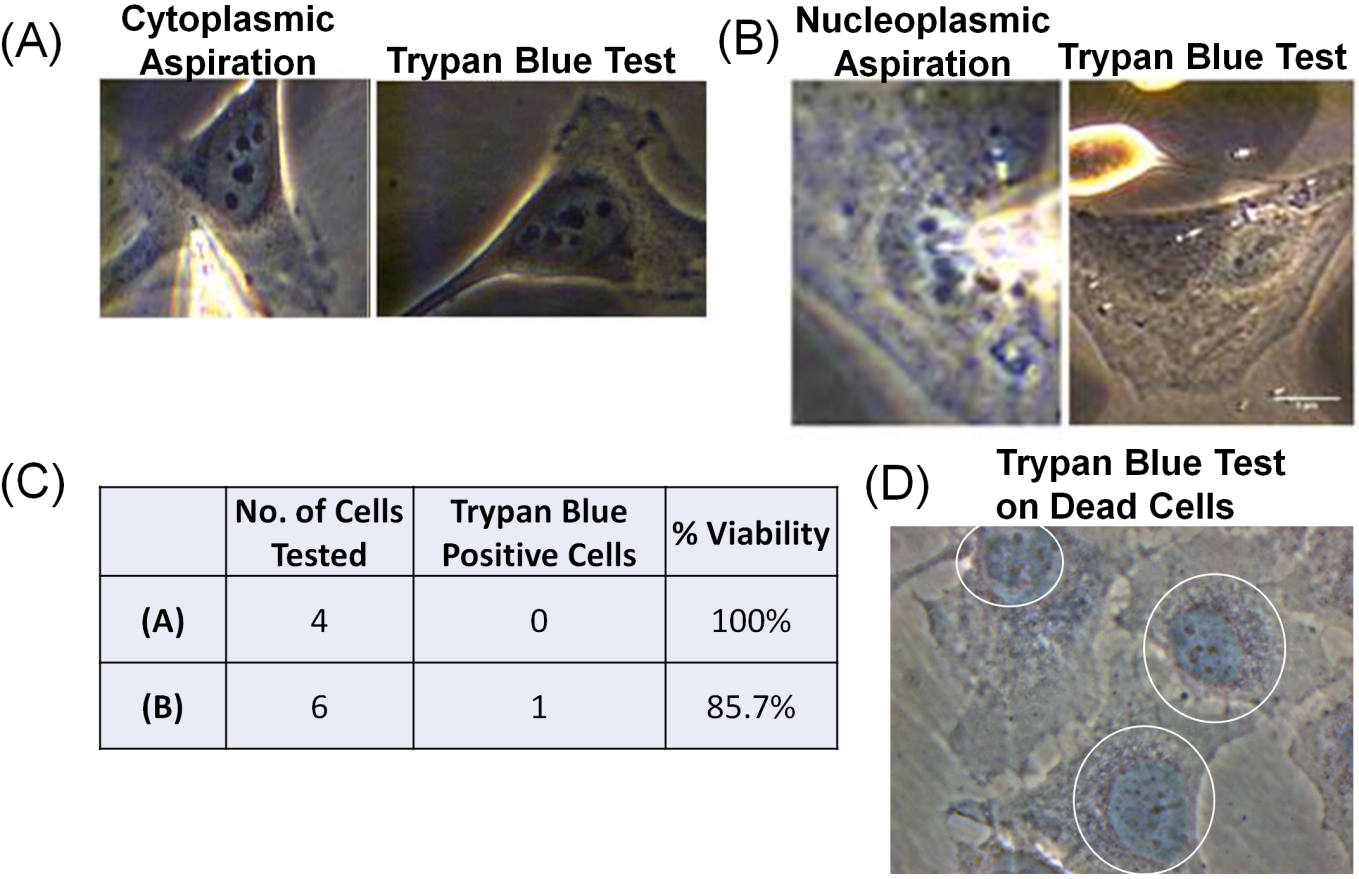
**

**Figure S2: Cell viability followed by nanoaspiration.** (A) Cytoplasmic or (B) nuclear nanoaspiration from HeLa cells was followed by staining of the same cell with a live/dead cell stain, trypan blue. Trypan blue stained or unstained cells look identical, suggesting the maintenance of complete cell viability. (C) Viability percentages were calculated to be 100% and 85.7% for cytoplasmic and nuclear aspirations, respectively. (D) Dead cells that take up the trypan blue stain more readily are shown alongside for comparison purposes.


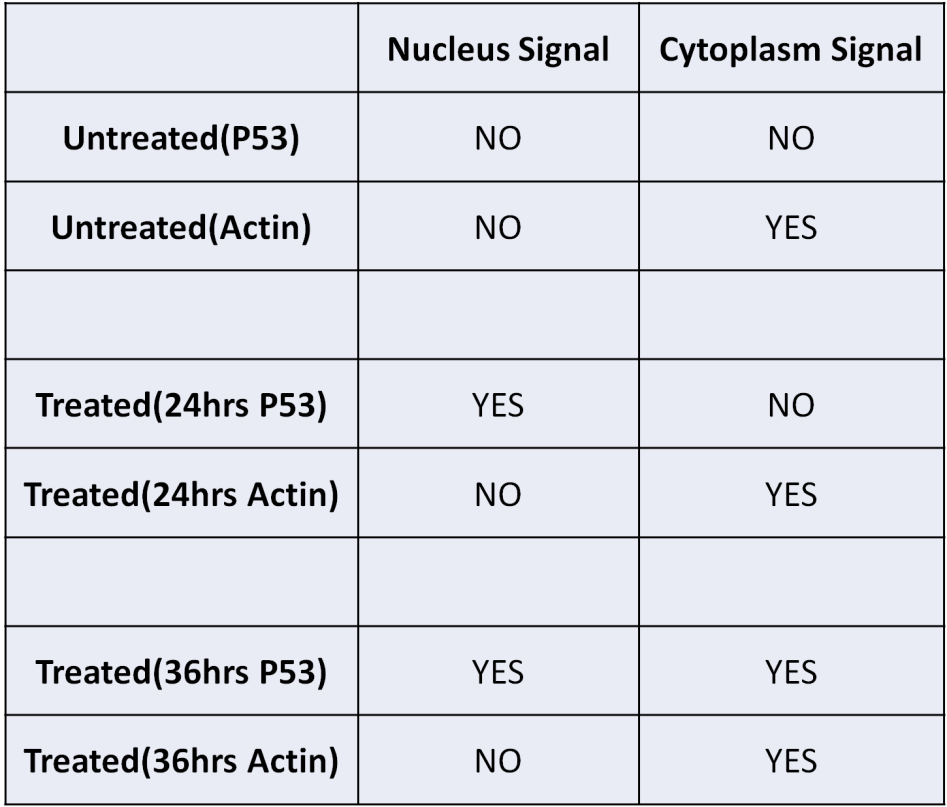


**Table S1: Protein detection from drug-treated cells.** Longitudinal sampling resulting in the positive or negative detection of β-actin and p53 proteins, in cytoplasmic or nuclear extracts from Actinomycin D-treated or untreated HeLa cells.
